# Comparison of methods for detecting mandibular lingula and can antilingula be used in lingula mandibula detection?

**DOI:** 10.1186/s12903-025-05788-8

**Published:** 2025-03-26

**Authors:** Emre Balaban, Taha Emre Köse, Dilara Nil Günaçar, Muhammed Enes Naralan, Merve Gonca

**Affiliations:** 1 Faculty of Dentistry, Department of Oral and Maxillofacial Surgery, Recep Tayyip Erdogan University, Rize, Turkey; 2 Faculty of Dentistry, Department of Oral and Maxillofacial Radiology, Recep Tayyip Erdogan University, Rize, Turkey; 3 Faculty of Dentistry, Department of Orthodontics, Eskisehir Osmangazi University, Eskisehir, Turkey

**Keywords:** Lingula, Antilingula, Inferior vertical ramus osteotomy, Bilateral sagittal split ramus osteotomy, Iatrogenic nerve injury

## Abstract

**Objective:**

The aim of this study is to evaluate the relationship between anatomical reference points used during orthognathic surgery and to minimize the risks of iatrogenic neurovascular damage.

**Materials and methods:**

This retrospective study included cone-beam computed tomography (CBCT) images involving the mandible from patients who visited Recep Tayyip Erdoğan University Faculty of Dentistry between January 2018 and September 2023. The age range of the included individuals was set between 18 and 80 years. Horizontal and vertical distances between mandibular anatomical structures, such as the lingula mandibula (LM), mandibular foramen (MF), antilingula (AL), and surrounding structures were measured using CBCT software. Individuals with intraosseous pathology, insufficient image quality, or a history of surgical/orthodontic treatment were excluded from the study.

**Results:**

A total of 240 hemimandibles from 120 patients were analyzed (55.83% female, 44.17% male; mean age: 46.78 ± 15.30 years). Significant differences were identified in LM positions according to different AL types. The LM was found to be more inferior and posterior relative to hill and ridge type ALs, while it was more anterior relative to plateau type ALs. In 26.25% of mandibular rami, AL was not detected.

**Conclusion:**

The position of the AL can serve as a guide in determining the osteotomy line during inferior vertical ramus osteotomy (IVRO). However, relying solely on AL as a reference point may increase the risk of inferior alveolar nerve (IAN) injury. Preoperative tomographic evaluations to determine the relationships among LM, MF, and AL can provide a safer approach in surgical planning, reduce complications, and help protect neurovascular structures.

## Introduction

In routine dental practices, particularly during procedures such as inferior alveolar nerve (IAN) block anaesthesia and orthognathic surgeries, including bilateral sagittal split ramus osteotomy (BSSO) and inferior vertical ramus osteotomy (IVRO), understanding the anatomical positions of the lingula mandibula (LM) and mandibular foramen (MF) is essential for preserving this neurovascular bundle. The IAN, the thickest branch of cranial nerve (CN) V3, enters the mandible through the MF, which is located on its medial surface. The lingula is an irregularly shaped bony prominence on the medial aspect of the mandibular ramus near the MF [1].

BSSO is the most commonly used method in mandibular orthognathic surgeries. The osteotomy should be performed horizontally, just above the LM located on the medial surface of the mandible. The most frequent complications of BSSO include IAN injury and improper fractures of the mandible. Permanent damage to the IAN is the complication that most significantly impacts a patient’s daily life [1–4]. The primary cause of this complication is performing the osteotomy line below the recommended level. Osteotomies conducted below this level can result in bleeding and neurological complications due to damage to the alveolar neurovascular bundle [4].

IVRO originally performed using an extraoral approach, has been conducted intraorally for over 30 years following the introduction of electric oscillating saws [5]. Despite being an older technique, IVRO is still widely used to treat mandibular prognathism [6]. It has been reported that IVRO procedures have a lower incidence of permanent neurosensory disturbances than BSSO procedures [7]. Additionally, IVRO has a shorter operative time compared to BSSO [7]. However, since this technique is performed on the lateral surface of the mandible, the medial structures, including the IAN, LM, and MF, cannot be directly visualized, presenting a disadvantage [8]. Therefore, an anatomical reference point on the lateral surface of the ramus was identified, specifically the most prominent bony point below the sigmoid notch (SN), which is referred to as the antilingula (AL) [9].

This study aims to determine the correlation between reference points used in different techniques during orthognathic surgery and to minimize the risks of iatrogenic neurovascular damage.

## Material and method

This study was conducted retrospectively by reviewing the archive records of cone-beam computed tomography (CBCT) images involving the entire mandible, obtained for various reasons (resorption, trauma, impacted teeth, etc.) from patients who visited the Department of Oral and Maxillofacial Radiology at Recep Tayyip Erdoğan University Faculty of Dentistry for examination between January 2018 and September 2023. Institutional research ethics approval was obtained from the Recep Tayyip Erdoğan University Non-invasive Clinical Research Ethics Committee (serial number: E-40465587-050.01.04-1227). The study adhered rigorously to the principles outlined in the Declaration of Helsinki throughout all stages of the research process. Informed consent forms were obtained from all patients. The age range of the individuals included in the study was determined to be between 18 and 80 years.

### Exclusion Criteria

1. Individuals with a history of intraosseous pathology (cyst/tumour) and/or syndromes (e.g., cleft palate or syndromes causing maxillary defects).
2. CBCT images that do not possess adequate diagnostic quality.
3. Cases with a history of surgical or orthodontic treatment.

CBCT images obtained using the NewTom VGI Evo (Cefla, Verona, Italy) device were evaluated using the image processing software Planmeca Romexis 4.6.2.R (Planmeca Romexis, Helsinki, Finland), and measurements were also performed using this software. CBCT imaging was performed with the following parameters: field of view (FOV) of 16 × 16 cm, tube voltage of 110 kVp, tube current of 3.00–3.85 mA, exposure time of 1.8 s.

During the scanning process, the patient’s age, gender, and the presence or absence of teeth in the evaluated region were recorded. Standardization in the evaluations has been ensured by aligning the sagittal plane perpendicular to the ground plane and the Frankfurt horizontal plane parallel to the ground plane. The right and left mandibular ramus were assessed separately on 3D images obtained from multiplanar reconstruction views.

**Fig. 1:**
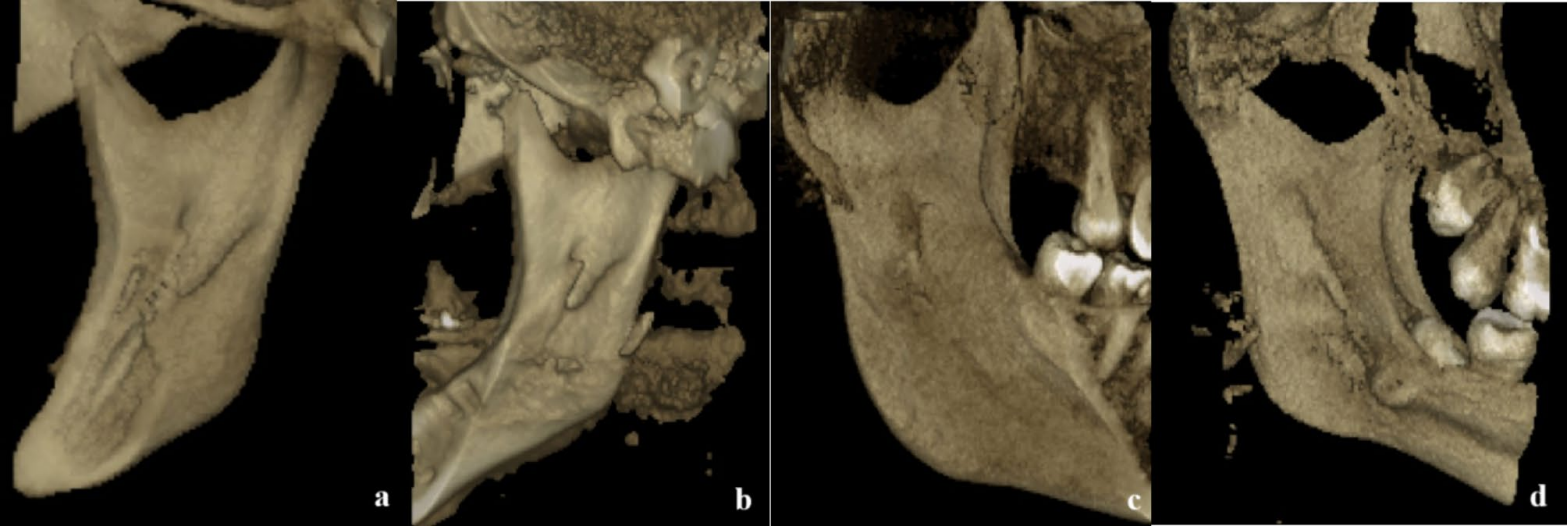
Different shapes of LM (**a**) Nodular; (**b**) Triangular; (**c**) Truncated; (**d**) Assimilated

**Fig. 2:**
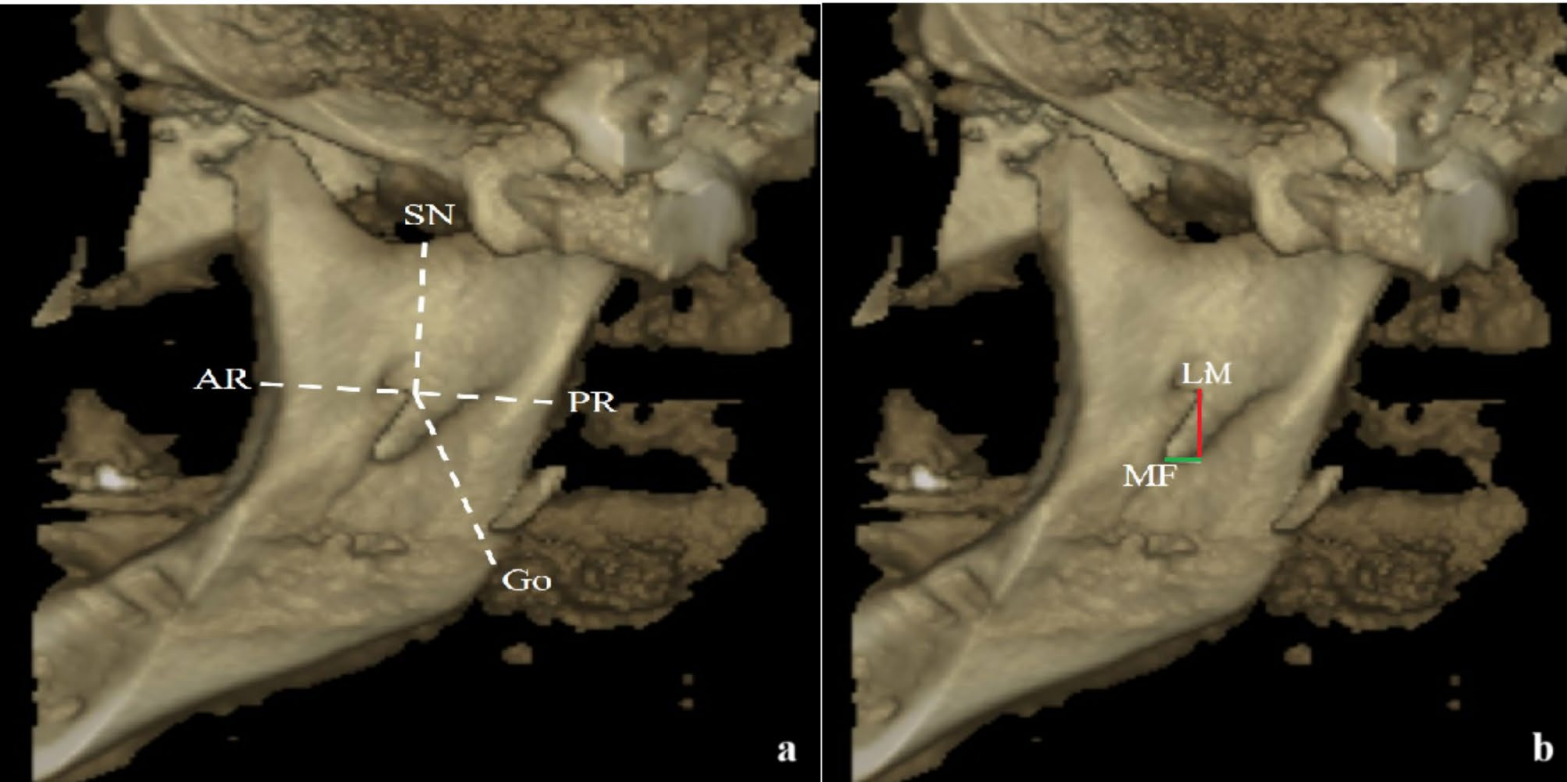
(**a**) Lingula Mandible measurements; (**b**) The vertical (red) and horizontal (green) distances between the lingula mandible and the mandibular foramen SN: Sigmoid Notch; AR: Anterior Ramus; PR: Posterior Ramus; Go: Gonion; LM: Lingula Mandible; MF: Mandibular Foramen

**Fig. 3:**
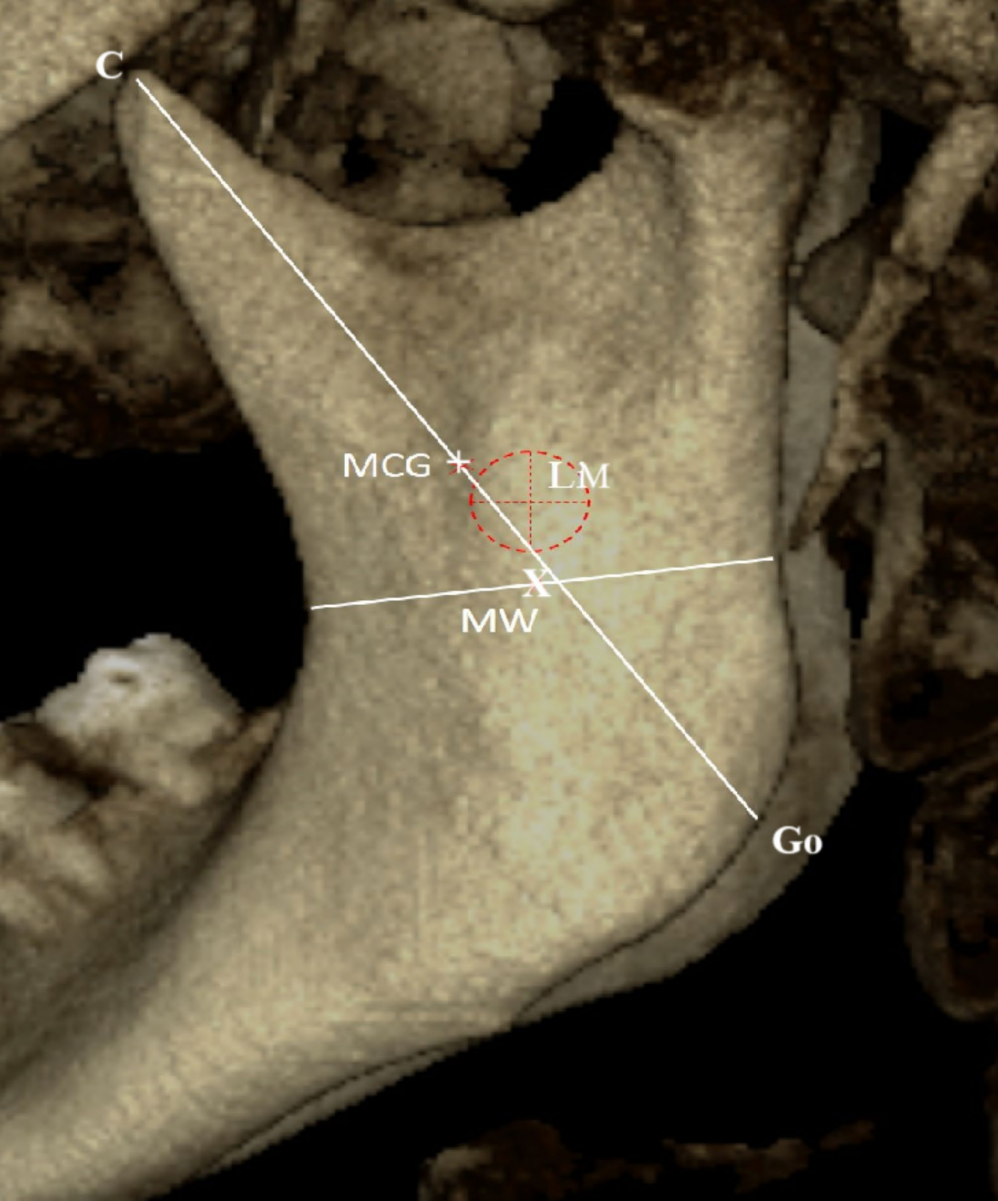
Marking on the external surface of the mandibular ramus showing the corresponding position of the tip of Lingula (x), the antilingula (+), the midwaist of the mandibular ramus (MW), and the midpoint between the coronoid process (C) and the gonion (Go) (MCG)

**Fig. 4:**
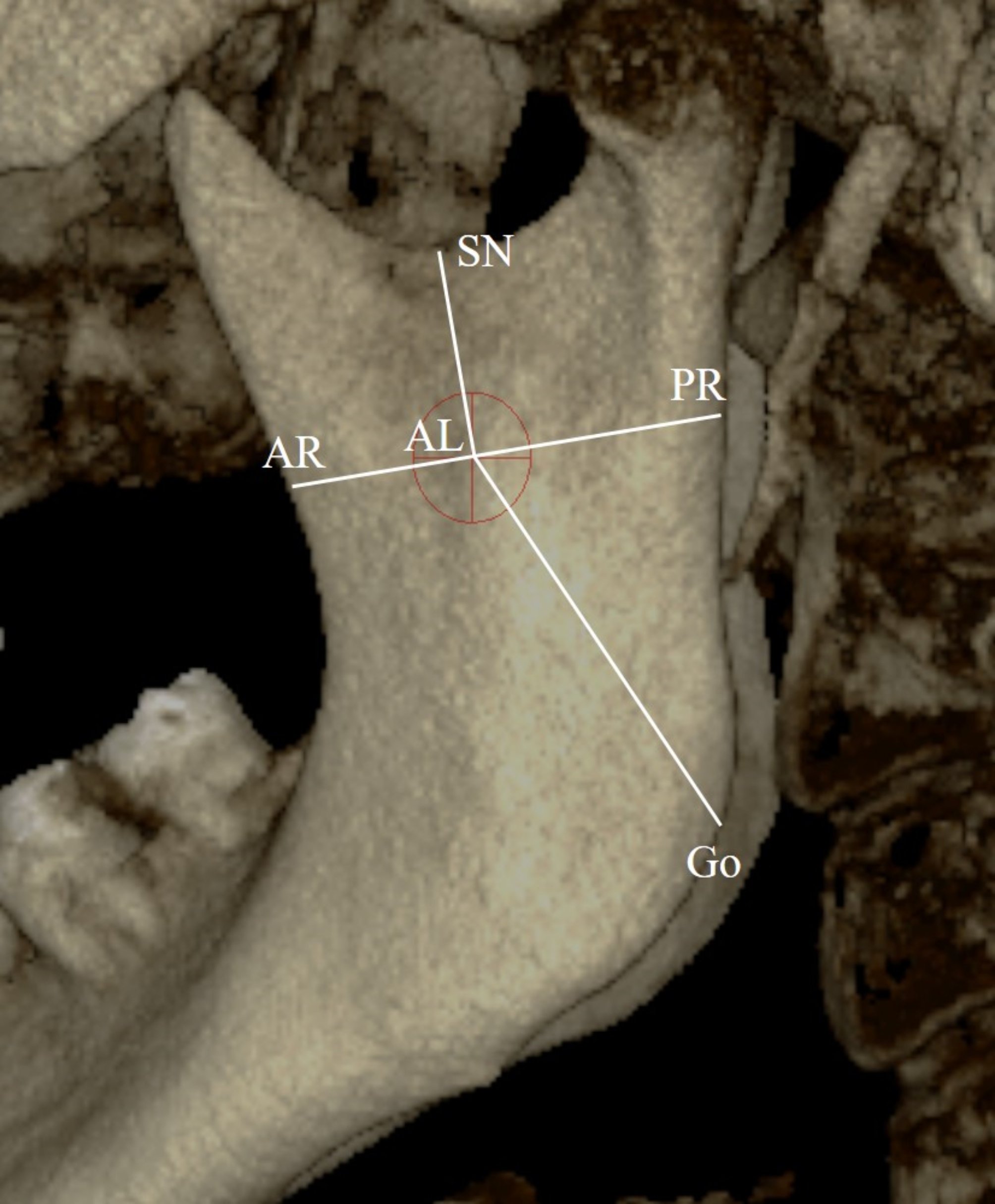
Antilingula measurements AL: Antilingula; SN: Sigmoid Notch; AR: Anterior Ramus; PR: Posterior Ramus; Go: Gonion

1. The lingula mandibula (LM) type was classified similarly to the study by Tuli et al. [10] as nodular (lingula was nodular and of variable size, almost the entire lingula except for its apex which was merged into the ramus), truncated (lingula with somewhat quadrangular bony projection on its top), triangular with a wide base and a narrow rounded or pointed apex, rounded or pointed apex), or assimilated (Fig. 1). The assimilated type indicates the absence of the LM.

2. The distances of the LM and mandibular foramen (MF) to the sigmoid notch (SN), anterior ramus (AR), posterior ramus (PR), and gonion (Go) were measured using the methods described by Sinanoğlu et al. [11] and Findik et al. [12]. Additionally, the vertical and horizontal distances between the MF and LM were measured as outlined in Sinanoğlu et al. [11] study (Fig. 2).

3. Horizontal and vertical measurements from the midpoint between the coronoid process and gonion (MCG) to the LM, as well as from the midpoint of the mandibular ramus (MW) to the LM, conducted similarly to the methods used by Apinhasmit et al. [13] (Fig. 3).

4. If antilingula (AL) is present, the distances of the AL to the SN, AR, PR, and Go measured following the methodologies of Chen et al. [14] and Findik et al. [12] (Fig. 4).

### Evaluations on the Lateral Surface of the Mandible

**Fig. 5:**
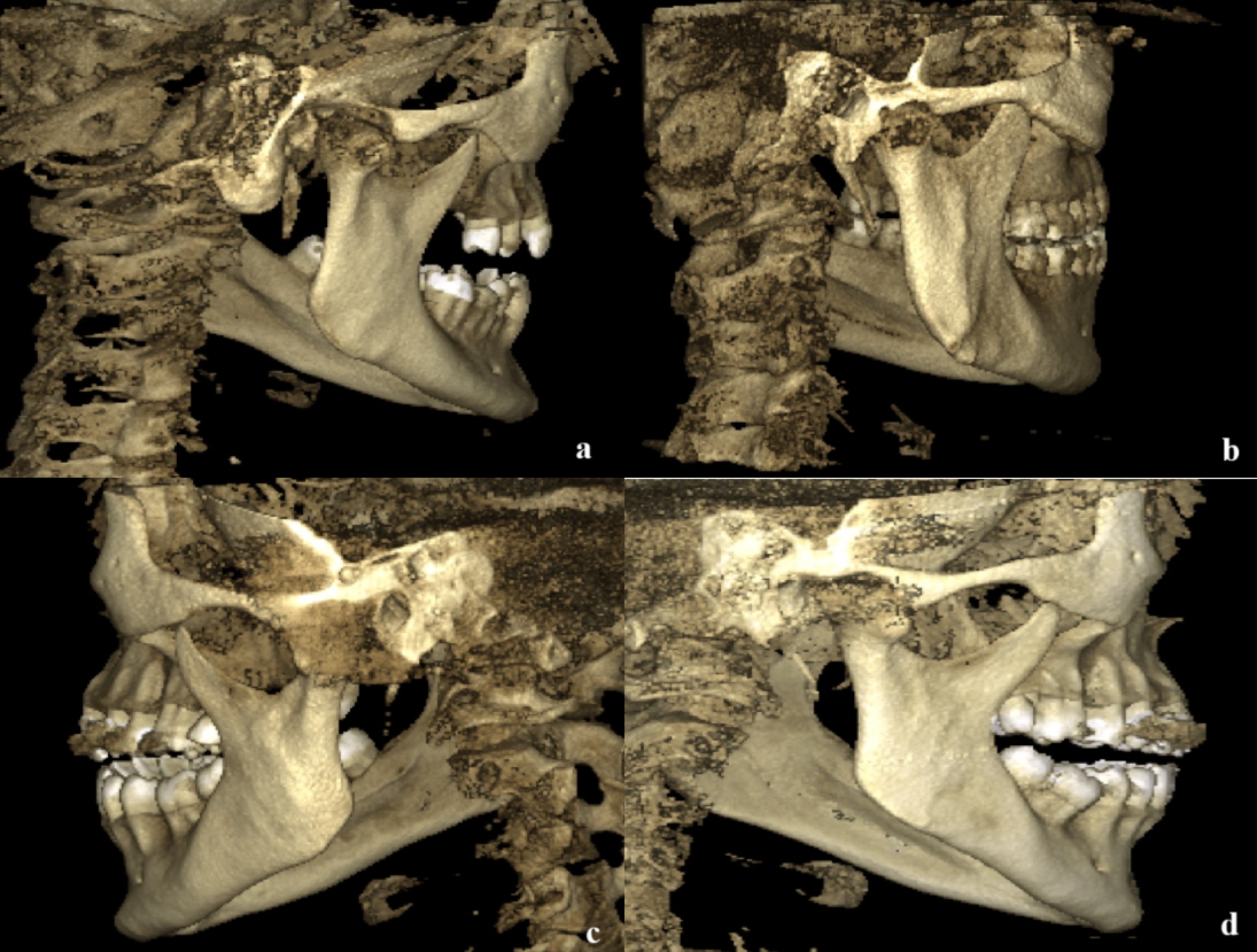
In the lateral aspect of mandibular ramus, four shapes of antilingula were classified. (**a**) Hill; (**b**) Ridge; (**c**) Platau; (**d**) Plain

**Fig. 6:**
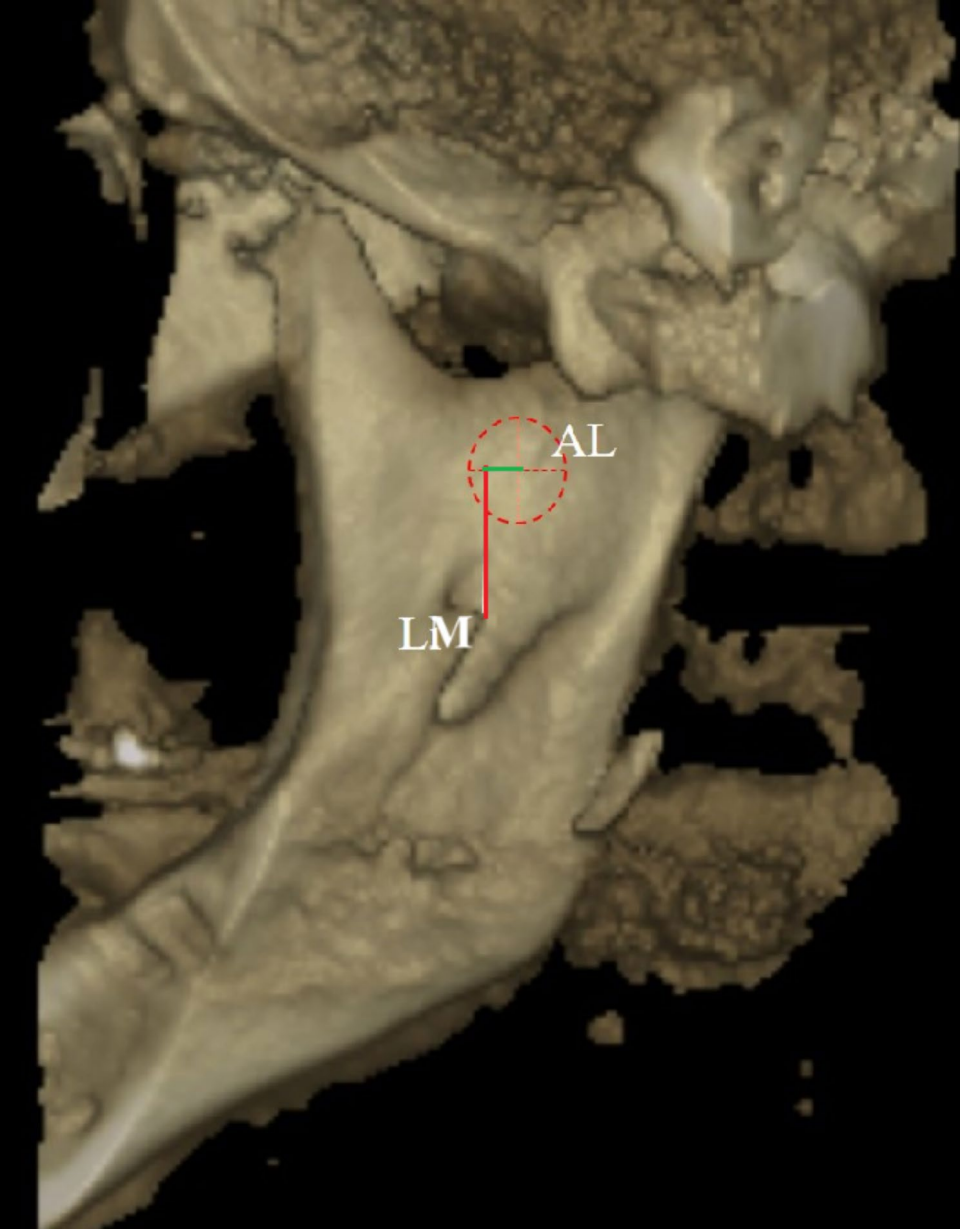
The vertical (red) and horizontal (green) distances between the lingula and antilingula LM: Lingula Mandible; AL: Antilingula

1. The AL type was classified similarly to the study by Chen et al. [14] as hill, ridge, plateau, or plain. A ‘hill’ that is observed to be higher than the surrounding area, a ‘ridge’ that has a narrow and raised part, and a ‘plateau’ a large, flat area that is higher than around it was evaluated as. An AL described as “plain” indicates the absence of antilingula (Fig. 5).

2. In the presence of both AL and LM, vertical and horizontal measurements between the two performed similarly to the study conducted by Sinanoğlu et al. [11] (Fig. 6).

### Statistical analysis

The sample size was calculated using G*Power 3.1 software (Heinrich-Heine University of Dusseldorf, Germany). A post hoc power analysis was conducted using a one-way analysis of variance (ANOVA) with a 95% confidence level (1-α), effect size (d) = 0.3877551, number of groups = 4, and a sample size of 120. The power was calculated as 95.0% test strength (1-β) [15].

Descriptive statistics were calculated and presented as mean and standard deviation (SD). The normality of data distribution was assessed using the Kolmogorov-Smirnov test. One-way ANOVA was used to determine the differences among groups in the parametric data obtained. The homogeneity of variance was evaluated using Levene’s test, and the post-hoc Tukey (Tukey HSD) test was applied for paired comparisons. For non-parametric data, the Kruskal-Wallis test was used. A *p*-value of < 0.05 was considered statistically significant.

A radiolog (T.E.K.) with 10 years of expertise performed all measurements. After 1 month, the same examiner (T.E.K.) re-analyzed 60 randomly chosen ramus to assess measurement errors.

## Results

The intraobserver agreement was estimated using the intra-class correlation coefficient (ICC) and was found to be excellent for all measurements (ICC ≥ 0.997).

This study was conducted on 120 patients, encompassing a total of 240 hemimandibles. The participant group consisted of 55.83% females and 44.17% males. In terms of age distribution, the mean age of the participants was calculated as 46.78 ± 15.30 years. When the classification was made according to lingula mandible types, nodular type LM was detected as 48.75% (117/240), truncated type LM as 16.25% (39/240), triangular type as 10.42% (25/240), and assimilated type as 24.59% (59/240).

Table 1 shows the positions of the lingula according to the types of antilingula. A significant difference was observed in the LM-SN distance. According to the table, patients with hill, ridge and plateau types antilingula had their lingula positioned more superiorly. A significant difference was also found in the distance between the lingula and the anterior ramus across antilingula types. This difference was observed to be more posterior in patients with plateau type antilingula.

**Table 1:** Lingula measurements based on antilingula types

	a type (*N* = 117 )		b type (*N* = 39)		c type (*N* = 25)		d type (*N* = 59)			post-hoc					
Lingula maesurements	Mean	SD	Mean	SD	Mean	SD	Mean	SD	*p* value	a-b	a-c	a-d	b-c	b-d	c-d
MF-GO ^b^	20.6	4.0	20.5	4.4	20.7	2.9	21.3	3.7	0.594						
MF-AR ^a^†	15.9	2.1	16.7	2.6	17.0	3.2	15.5	2.7	0.025						
MF-PR ^b^	14.0	2.1	13.2	1.9	13.9	2.7	14.5	2.4	0.105						
MF-SN ^a^†	22.5	4.1	23.2	3.9	22.6	4.1	23.8	4.4	0.245						
LM-GO ^b^	27.3	4.2	27.8	5.0	27.7	4.6	27.4	4.3	0.933						
LM-AR ^a*^ (tukey)	15.8	2.4	16.5	2.2	16.8	2.9	14.9	2.2	**0.001**	0.351	0.186	0.112	0.949	**0.008**	**0.005**
LM-PR ^b^	14.4	1.8	13.9	2.1	14.5	2.3	14.8	2.1	0.340						
LM-SN ^a*^ (tukey)	14.2	3.2	14.3	2.9	14.3	2.9	16.6	3.5	**< 0.001**	1.000	1.000	**< 0.001**	1.000	**0.004**	**0.018**
LM-MF VERTİKAL ^b^	8.6	2.3	9.2	2.5	11.1	12.8	8.0	2.9	0.328						
LM-MF HORİZONTAL ^b^	1.7	1.2	2.0	1.5	1.4	0.9	1.6	1.2	0.188						

MF: Mandibular Foramen; Go: Gonion; AR: Anterior Ramus; PR: Posterior Ramus; SN: Sigmoid Notch; LM: Lingula Mandible; a type: hill antilingula; b type: ridge type antilingula; c type: plateau type antilingula; d Plain type antilingula

a One-way analysis of variance (ANOVA). *p*<0.05.

No significant difference was found between the different antilingula types (Table 2).

**Table 2:** Antilingula measurements based on antilingula types

	a type (*N* = 94)		b type (*N* = 45)		c type (*N* = 38)		
Antilingula measurements	Mean	SD	Mean	SD	Mean	SD	*p* value
AL-GO ^a^	30.3	4.8	30.8	5.2	31.1	5.7	0.715
AL-AR ^b^	16.5	7.3	16.0	2.7	17.0	3.7	0.213
AL-PR ^b^	15.2	2.7	18.9	24.2	14.2	2.5	0.062
AL-SN ^a^	12.6	4.0	13.4	3.6	12.6	4.0	0.477

AL: Antilingula; GO: Gonio; AR: Anterior Ramus; PR: Anterior Ramus; SN: Sigmoid Notch; a type: hill type antilingula; b type: ridge type antilingula; c type: plateau type antilingula

^a^One-way analysis of variance (ANOVA).

^b^Kruskal–Wallis test. *p* < 0.05

The vertical positions of the lingula in each antilingula type are shown in Table 3. According to these results, in all antilingula types, the lingula was located more inferiorly compared to the antilingula.

**Table 3:** Vertical relationship between antilingula

Vertical	Mean(mm)	SD(mm)	*n*
AL-LM	-1,79	4,29	176
a	-2,1	4,5	93
b	-1,2	3,9	45
c	-1,9	4,1	38
MW-LM	2,5	2,4	240
MCG-LM	-0,1	2,7	240

AL: Antilingula; LM: Lingula; MW: Midpoint of the mandibular ramus; MCG: Midpoint between coronoid process and gonion; a type: hill antilingula; b type: ridge type antilingula; c type: plateau type antilingula; SD: Standard deviation

Negative values indicate direction, specifically denoting the inferior orientation

The horizontal positions of the lingula in relation to the antilingula in each type are presented in Table 4. According to these findings, in hill and ridge type antilingula, the lingula was positioned more posteriorly relative to the antilingula, while in plateau type antilingula, the lingula was positioned more anteriorly.

**Table 4:** Horizontal relationship between antilingula and lingula

Horizontal	Mean(mm)	SD(mm)	*n*
AL-LM	-0,2	2,8	176
a	-0,1	2,7	93
b	-0,9	2,2	45
c	0,7	3,4	38
MW-LM	-0,6	1,9	240
MCG-LM	-3,9	2,3	240

AL: Antilingula; LM: Lingula; MW: midpoint of the mandibular ramus; MCG: Midpoint between coronoid process and gonion; a type: hill type antilingula; b type: ridge type antilingula; c type: plateau type antilingula; SD: Standart deviation

Negative values indicate direction, specifically denoting the posterior orientation

Antilingula was not detected in 26.25% of the mandibular rami, highlighting its absence in a significant portion of the study population. This finding reinforces the necessity of additional anatomical landmarks when planning osteotomy procedures.

## Discussion

In this study, where the position of the LM was investigated according to AL types, it was concluded that the position of the LM can be partially predicted based on the AL type. Specifically, in hill and ridge type ALs, the LM is positioned more inferior-posterior, whereas in plateau type AL, it is positioned more infero-anterior. However, no significant change in the position of the AL was found according to its type.

Aging is known to influence skeletal morphology, including mandibular structures. However, previous study suggest that the most notable age-related change in mandibular morphology is a reduction in symphysis height, while other parameters remain relatively stable [16]. In our study, despite the higher mean age of the participants, the mandibular morphology is still relevant for evaluation, as the structural integrity of the mandible remains largely preserved with age. This supports the validity of our study population and findings.

In previous studies, including the classification by Chen et al. [14], AL was categorized into four types: hill, ridge, plateau, and plain. However, in our study, we refined this classification by introducing subcategories of hill, ridge, and plateau type, while excluding plain type. The plain category AL contained cases that did not fit into any specific classification, potentially leading to inconsistencies. By eliminating this category, we aimed to create a more systematic and precise classification framework, allowing for a clearer interpretation of AL variations.

The MF is an opening on the medial surface of the mandibular ramus where the inferior alveolar neurovascular bundle enters the mandible. The edge of the MF typically has a “V” shape. The LM is a small bony prominence located just above the MF opening and is often used as an anatomical landmark for the IAN block. For a predictably successful IAN block, the needle tip must approach the MF and be at least 5 mm above the LM [17]. 

Park et al. [17] reported that the LM is located 0.8 mm anterior and 7.5 mm superior to the MF. However, other studies have indicated that the LM is positioned 7–7.8 mm superior to the MF [18, 19]. In this study, the vertical distance between the LM and MF, based on AL types, was found to be 8.6 mm, 9.2 mm, 11.1 mm, and 8.0 mm, respectively. The horizontal distance between the LM and MF, according to AL types, was 1.7 mm, 2.0 mm, 1.4 mm, and 1.6 mm, respectively. Therefore, in all AL types, the LM is positioned superior and anterior to the MF.

Another situation where the MF and LM are used as anatomical landmarks is during BSSO in orthognathic surgery. BSSO is one of the most frequently performed surgical procedures today, and the risk of IAN damage is a known complication [20]. The MF and LM are critical anatomical landmarks for medial surface horizontal osteotomies during BSSO. Numerous reports emphasize that the medial horizontal osteotomy should be performed just above the LM and extended as far back as possible from its posterior edge. However, identifying the LM can be challenging due to limited surgical visualization, muscle-tendon attachments in this area, and morphological variations [21].

The AL, which serves as the counterpart to the LM, is a small bony prominence located on the lateral side of the ramus. This prominence was identified by Aziz et al. [22] as an anatomical landmark to help prevent IAN damage due to the difficulty in accessing and identifying the LM from the buccal side of the ramus during IVRO. Reitzik et al. [23] proposed that the AL serves as the attachment site for the masseter muscle, describing this prominence as the masseteric apical protrusion. The way the masseter muscle attaches to the mandibular ramus and its strength can influence both the formation of the AL and the size of the protruding area. Therefore, the AL may not always be detectable.

An anatomical study examining the presence and position of the AL was conducted by Yates et al. [24]. This study, involving three researchers, analyzed 70 dried mandibles and found that the AL was present in only 44% of cases, with 15% showing complete absence and the remaining 41% categorized as “uncertain.” Additionally, they found that the position of the AL was highly variable in relation to the MF, with only 18% of cases showing a distance of 3 mm or less between these two landmarks.

Another anatomical study by Tamas, conducted on 200 rami, revealed that the AL could be definitively identified in only 54% of cases [25]. In our study, the AL was not identifiable in 63 rami (26.25%). Furthermore, hill type AL was detected in 94 rami (39.17%), ridge type AL in 45 rami (18.75%), and plateau type AL in 38 rami (15.83%).

Aziz et al. [22] reported that, in most cases, the LM is located below and behind the AL. Pogrel et al. [26] found that the likelihood of the LM being positioned below and behind the AL is 68.3%, with an average distance of 5.39 mm between them. Park et al. [17] demonstrated that, on average, the LM is positioned 4.19 mm posterior and 0.54 mm superior to the AL. Additionally, the MF is located 4.98 mm posterior and 6.95 mm inferior to the AL.

In our study, consistent with these findings, it was observed that in all AL types, the LM is positioned more inferiorly compared to the AL. In hill and ridge type ALs, the LM was found to be more posterior to the AL, whereas in plateau type AL, it was positioned more anteriorly.

Researchers [17, 27, 28] have suggested that the position of the osteotomy cut in IVRO can be determined based on the position of the AL, with the osteotomy line placed behind the AL to prevent damage to the IAN. However, relying solely on the AL as the primary reference point for determining the osteotomy line may increase the risk of IAN injury. Therefore, incorporating the positions of the LM and MF, particularly their anterior-posterior and superior-inferior dimensions, into the planning process could offer a safer approach when defining the osteotomy line for IVRO.

Currently, a standardized anatomical measurement specific to IVRO has not been established, making it inappropriate to use the AL as an absolute reference point during surgical procedures. Instead, preoperative tomographic evaluations assessing the relationships among the MF, LM, and AL positions with AL serving as a guiding reference for IVRO are crucial for safeguarding the IAN. This comprehensive approach could significantly reduce the risk of surgical errors and postoperative complications.

This study has several limitations that should be acknowledged. The retrospective nature of the study may introduce inherent biases related to data collection and patient selection. The study population did not consist of actual surgical patients, which may limit the direct clinical applicability of the findings to orthognathic surgery planning. All participants were from a single ethnic background, which may limit the generalizability of the findings to other populations.

Future studies with prospective designs, larger and more diverse patient cohorts, and multiple measurement sessions are needed to validate these findings and further refine the clinical implications of antilingula-based osteotomy guidance.

## Conclusion

According to the findings of this study, the placement of cuts during IVRO should be determined based on the position of the AL. In hill and ridge type ALs, the LM was observed to be positioned more posteriorly and inferiorly relative to the AL. Therefore, in these types, placing the osteotomy line immediately behind the LM and posterior to the AL is recommended to prevent IAN injury. Conversely, in plateau type AL, the LM was found to be located more anteriorly. In such cases, the osteotomy line should be placed toward the anterior portion of the AL, ensuring that the cuts are positioned further from the LM.

However, as AL was not detected in 26.25% of the mandibular rami, it cannot be considered a universal guide for the osteotomy line. Therefore, this approach should only be applied in where the specified AL types are clearly identified. Careful planning of the osteotomy line according to AL types remains essential, both to account for the LM’s position and to protect the IAN.

## Data Availability

The datasets used and/or analysed during the current study are available from the corresponding author on reasonable request.

## Abbreviations

AL: Antilingula
ANOVA: One-way analysis of variance
AR: Anterior Ramus
BSSO: Bilateral Sagittal Split Ramus Osteotomy
CBCT: Cone-Beam Computed Tomography
CN: Cranial Nerve
Go: Gonion
IAN: Inferior Alveolar Nerve
ICC: Intraclass correlation coefficient
IVRO: Inferior Vertical Ramus Osteotomy
LM: Lingula Mandibula
MCG: Midpoint between Coronoid Process and Gonion
MF: Mandibular Foramen
MW: Midpoint of the Mandibular Ramus
PR: Posterior Ramus
SD: Standard Deviation
SN: Sigmoid Notch
